# Improving Geodetic Monitoring in the Aeolian Archipelago: Performance Assessment of the Salin@net GNSS Network

**DOI:** 10.3390/s25237362

**Published:** 2025-12-03

**Authors:** Federico Pietrolungo, Alessandra Esposito, Giuseppe Pezzo, Aladino Govoni, Letizia Anderlini, Mirko Iannarelli, Andrea Terribili, Claudio Chiarabba, Mimmo Palano

**Affiliations:** 1 Sezione Osservatorio Nazionale Terremoti, Istituto Nazionale di Geofisica e Vulcanologia (INGV), 00143 Rome, Italy; alessandra.esposito@ingv.it (A.E.); giuseppe.pezzo@ingv.it (G.P.); aladino.govoni@ingv.it (A.G.); mirko.iannarelli@ingv.it (M.I.); andrea.terribili@ingv.it (A.T.); claudio.chiarabba@ingv.it (C.C.); 2 Sezione Bologna, Istituto Nazionale di Geofisica e Vulcanologia (INGV), 40128 Bologna, Italy; letizia.anderlini@ingv.it; 3 Dipartimento di Scienze della Terra e del Mare, Università degli Studi di Palermo, 90123 Palermo, Italy; mimmo.palano@unipa.it; 4 Sezione Osservatorio Etneo, Istituto Nazionale di Geofisica e Vulcanologia (INGV), 95125 Catania, Italy; 5 Consiglio Nazionale delle Ricerche, Istituto di Geologia Ambientale e Geoingegneria (CNR-IGAG), 00185 Rome, Italy

**Keywords:** GNSS network, GNSS quality, Aeolian Islands, GNSS in geodynamics, multipath effect

## Abstract

The Aeolian Archipelago, located in the southern margin of the Tyrrhenian Sea, is a key area to investigate the interplay between regional active fault systems and volcanic activity, making it a focal point for geodynamic studies. In particular, Salina Island lies at the intersection of two major tectonic structures: the Sisifo–Alicudi fault system in the western sector and the Aeolian–Tindari–Letojanni fault system in the central sector both exert a significant influence on the region’s deformation patterns. Detecting these signals requires high-quality GNSS data, yet the performance of newly installed stations in tectonic environments must be rigorously assessed. Between June 2023 and February 2024, a new continuous local GNSS network, which consists of five stations, Salin@Net, was established, on Salina Island. The central scientific objective of this study is to verify whether the new GNSS network achieves the data quality necessary for reliable geodetic monitoring and to evaluate its potential to resolve strain gradients in the area. We performed an extensive performance analysis of Salin@net GNSS stations, analyzing data quality, encompassing assessments of multipath effect, signal-to-noise ratio, observation continuity, and cycle slip occurrences, alongside GNSS position time series. These metrics were compared against the ISAL-RING station and benchmarked International GNSS Service (IGS) standards. Results show that the newly installed stations consistently meet the required standards, delivering robust and reliable measurements that are comparable to those of the RING GNSS continuous network. Positioning time series, processed in the ITRF14, indicate that the precision of the derived velocity estimates is comparable to that of standard continuous stations, although longer time spans are required to better constrain linear velocity estimates. Finally, spherical wavelet analysis demonstrates that the geometry of Salin@net significantly improves the spatial resolution of the strain field across the Aeolian–Tindari–Letojanni fault system and enhances resolution along the Sisifo–Alicudi fault, underscoring the role of dense, small-aperture GNSS networks in tectonic environment.

## 1. Introduction

The Aeolian Archipelago is the emerging portion of a ring-shaped volcanic complex on the southern border of the Tyrrhenian Sea [1]. Its evolution is linked to the Africa–Eurasia convergence, which has been active since the Cretaceous [2] and occurring at rates of 1–2 cm/yr [3].

**Figure 1:**
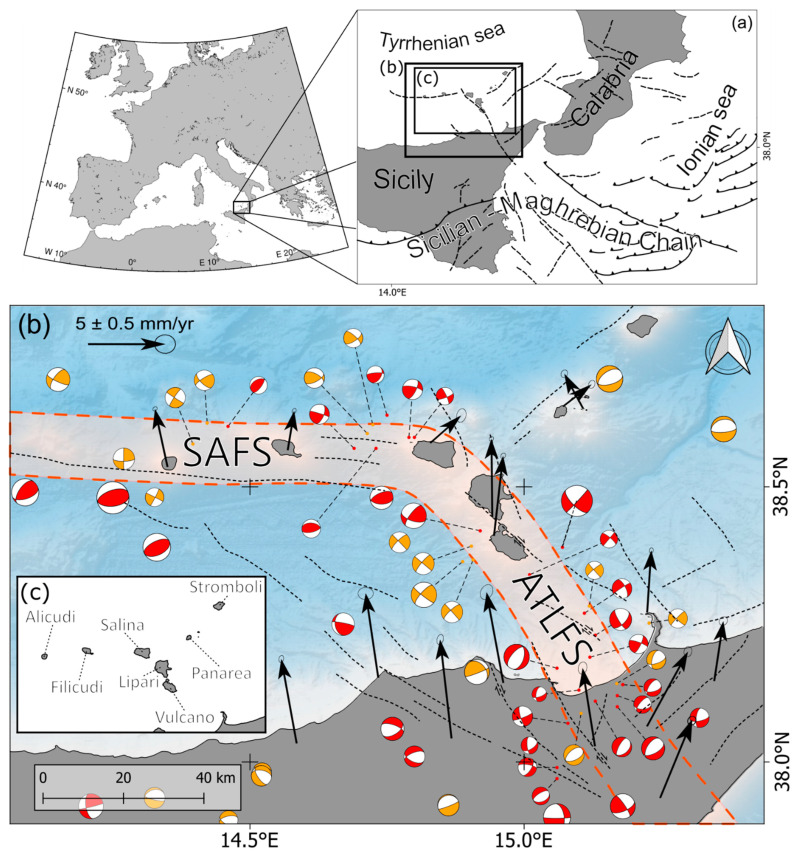
(**a**) Simplified tectonic map of Sicily and Calabria and surroundings; (**b**) fault systems, principal tectonic lineaments (SAFS, Sisifo–Alicudi Fault System; ATLFS, Aeolian–Tindari–Letojanni Fault System) from Palano et al. [6]. The black crosses represent the intersections between the reference meridians and parallels. Fault plane solutions are, from 1977 up to 2011, from Presti et al. [15] (compressive quadrants in orange) and, from 2012 up to 2024, TDMT [16] (compressive quadrants in red). GNSS velocity field (ETRF2014 [17]) is from the RING database [13] (update 1 December 2023); (**c**) schematic map for Aeolian Islands.

Slab rollback and back-arc extension have governed the tectonic development of the region, driving the formation of new oceanic crust in the Tyrrhenian basin and promoting slab tearing [4,5]. A set of recent studies suggests that the Sisifo–Alicudi and Aeolian–Tindari–Letojanni fault systems, developed around 2 Ma and 0.8 Ma, respectively [6], may be related to tearing processes in the Calabrian Arc [7]. The nature and extent of the interaction between the Sisifo–Alicudi and Aeolian–Tindari–Letojanni fault systems remain poorly understood. One of the main differences between them lies in their kinematics as seismic data evidence: the Sisifo–Alicudi fault system is characterized by compressive deformation whereas the Aeolian–Tindari–Letojanni fault system exhibits from transpressive to transtensive right-lateral deformation. Seismic events occurring since 1980 (Figure 1) show a predominance of thrust mechanisms with E-W-oriented planes along the Sisifo–Alicudi fault system, and primarily strike-slip mechanisms trending from NNW-SSE to NW-SE along the Aeolian–Tindari–Letojanni fault system [8,9].

Seismic data evidence is supported by geodetic data, which show a velocity decreasing in the north–south direction of the GNSS stations (Figure 1) along the Sisifo–Alicudi fault system, while the GNSS velocities along the Aeolian–Tindari–Letojanni fault system are more likely coherent with the right-lateral strike-slip motion of the eastern sector of the Aeolian Archipelago, with respect to the western sector (Figure 1). The authors identified a zone of transtensional deformation associated with the NNW-trending Aeolian–Tindari–Letojanni fault system, marking the transition from compressional to extensional tectonics across the central Aeolian sector [10,11]. More recent morphotectonic studies [12] further highlight the presence of a broad right-lateral deformation zone immediately south of Vulcano Island.

In this study, we present the newly established continuous GNSS network, Salin@net, specifically designed to monitor ground displacements across Salina Island and able to provide the high-resolution data needed to understand the complexities of this crucial tectonic junction.

To capitalize on this enhanced observational capability, the next section details the Salin@Net configuration—station locations, monumentation, instrumentation, and quality of data—and explains how the new network is integrated with the long-standing ISAL site and with regional GNSS arrays from the RING network [13].

To validate the performance of the new network, we assessed the quality of the geodetic observations collected by the five newly installed GNSS stations on Salina Island. We evaluate key parameters including the number of observations, multipath, the number of cycle slips, the signal-to-noise ratio, and the density of observations on a skyplot. Parameters are compared to ISAL GNSS station and “The Guidelines for Continuous Reference Stations” manual, published by the International GNSS Service (IGS) [14]. Additionally, we analyzed the updated GNSS spatial geometry using spherical wavelet decomposition to assess the contribution of the Salin@net network in detecting strain partitioning along the Sisifo–Alicudi and Aeolian–Tindari–Letojanni fault systems.

## 2. Geodetic Monitoring Background

**Figure 2:**
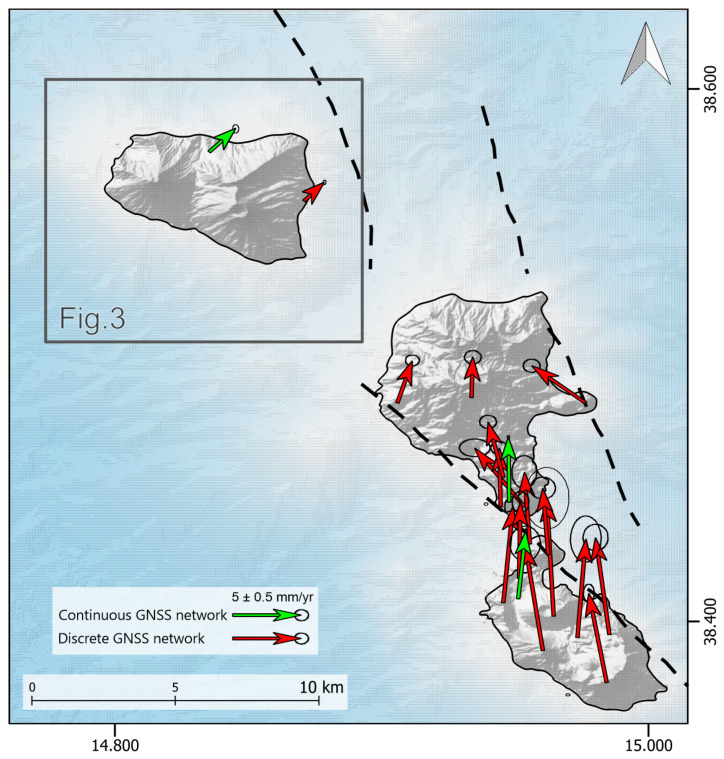
Compiled GNSS velocity field (ETRF2014 [17]) with solutions of continuous GNSS network (green) from the RING database [13] and discrete GNSS network (red) from Esposito et al., [21]. Dashed black lines represent principal tectonic lineaments from Palano et al. [6].

The first GPS survey, by single-frequency receivers, was conducted in 1987 as part of the Wegener/Medals-Calabrian Arc project [18]. The subsequent upgrade to dual-frequency GPS significantly enhanced positioning accuracy. Starting from 1991, under the Tyrgeonet project [19], several continuous GPS stations were installed to monitor tectonic deformation in the region [20]. Between 1995 and 2015, repeated GPS campaigns were carried out across the Aeolian Archipelago [21] (Figure 2), with observations conducted annually or every two to three years. The AINET-GPS repository, accessible to the scientific community since 2015, hosts GNSS campaign data archive collected between 1995 and 2013 (free access via anonymous FTP at ftp://ftp.ingv.it/pro/ainet-gpsdata/ (accessed on 12 March 2025)).

Continuous GNSS monitoring in the Aeolian Archipelago is currently overseen by the INGV RING network [13], including one continuous GNSS station per island. Beyond INGV, on Salina Island, the SALI GNSS station was set up at the municipal building in Santa Marina di Salina as part of the Tyrgeonet Project [19], collecting data during measurement campaigns from 1995 to 2013 [21]. From 2018, the site was equipped with a permanent station providing continuous data and the site was renamed to ISAL. It is currently active and data are available at the RING portal. On the northern face of Salina Island, the LINA continuous GNSS station, installed in 2014 and part of the NETGEO network by TOPCON, is also currently active and data are available on EPOS database [22].

In addition to GNSS measurements, several geodetic complementary techniques are being investigated for ground deformation analysis. In particular, InSAR products have been widely assessed to determine which MT-InSAR approaches are most suitable for detecting deformation in small island environments [23,24].

## 3. Methods

### 3.1. Salin@net

To adequately measure both long-term and short-term deformations and contribute to the investigation of the ATLF, we established, beginning from June 2023, a dense GNSS network on Salina Island. The network geometry was designed to provide a quite uniform coverage of Salina Island, taking into account the availability of other continuous stations managed by public institutions. A preliminary list of potential sites was defined and carefully inspected to evaluate the presence of possible obstructions. The GNSS stations (IS01, IS02, IS03, and IS04) were installed at distances ranging from 1.8 to 4 km from each other on the roofs of public buildings, the latter of which were selected after thorough checks of their structural solidity and integrity. This configuration provides convenient access to a continuous electricity supply. The IS05 station was installed on bedrock outcrops on the southwestern slope of “Mount Porri” and powered by a set of batteries and solar panels. Stations IS01, IS02, IS03, and IS04 were installed in late June 2023, while IS05 was installed in mid-January 2024. Stations coordinates and associated equipment are reported in Table 1. The network was named “Salin@net”.

The established GNSS stations have been equipped with STONEX instrumentation (e.g., STONEX (Paderno Dugnano, Milano, Italy) STXSA1100 antennas and SC600+ receivers), installed on 40 cm rods anchored to the rooftop of each building. IS05 was installed on a 65.1 cm rod anchored directly to the bedrock. Each station was equipped with a mobile internet connection so that the acquired data were directly transmitted to the INGV database in the proprietary raw format. The data acquisition sampling rate was set to 30 s.

### 3.2. Quality Parameters

A crucial step in assessing both the proper installation of GNSS stations and the future reliability of derived results is the analysis of observation quantity and quality within the RINEX files. Although many error sources such as receiver and satellite clock offsets and ionospheric and tropospheric delays [25] are external to the installation itself, several installation-related metrics can be directly quantified from RINEX data. These include the total number of observations, multipath indicators, cycle slip occurrences, signal-to-noise ratios, and sky-view plots. A proper installation will yield a continuous, multi-frequency observation record, free of obstructions, reflective surfaces, or radio-frequency interference at the antenna site [26].

The integration of multi-frequency and multi-system data is essential for achieving high-accuracy positioning. Several software packages for evaluating RINEX-based data quality exist. TEQC [27] works only with RINEX 2.xx files, while BNC (https://igs.bkg.bund.de/ntrip/bnc (accessed on 12 March 2025)) does not support BDS satellites. We opted for ANUBIS [28], a versatile and freely available tool, able to analyze RINEX 3.xx and 2.xx files across all constellations.

**Figure 3:**
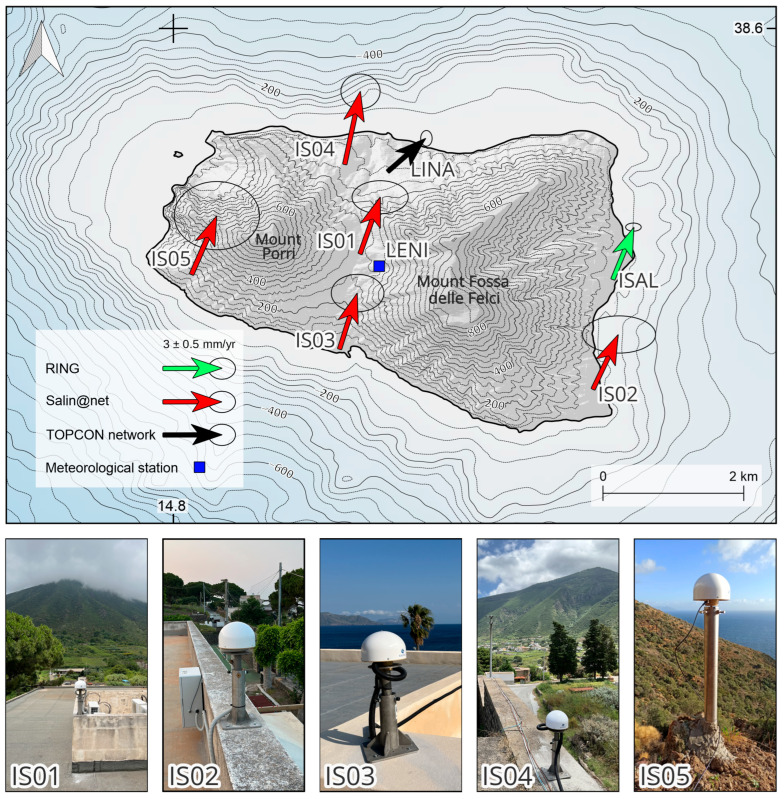
Velocity field of Salin@net. Photos of GNSS monuments are reported.

We used RINEX data (Table 1) and navigation files (mixed constellations from https://cddis.nasa.gov/index.html (accessed on 12 March 2025)) as input and obtained XTR files as output, which is the format used by ANUBIS 3.10 to describe observations quality. Our study evaluated data quality by comparing our results with the “Guidelines for Continuously Operating Reference Stations” from the International GNSS Service [14] and, as previously mentioned, data from ISAL station, part of the RING network [13]. ISAL, installed in 2015 with a LEICA GR25 receiver, provided a well-suited reference for assessing the quality of data collected by Salin@net. To enhance data visualization, we developed custom software that produces time series plots of station performance over an analyzed time period. This method provides critical insights into the efficiency of new installations, particularly during the early years of satellite data acquisition. Moreover, we compared a set of parameters with respect to the ones estimated from a nearby continuous station equipped with standard-cost instrumentation (e.g., ISAL; Figure 3 and Table 1). It is important to note that the ISAL GNSS station has not always recorded data in the RINEX 3.xx format. Before 25 June 2024, it only provided RINEX 2.11 files. For this reason, the quality parameters discussed has been considered only when the calculated or observed parameters are available for all stations. As shown in Table 1, the ISAL station—unlike the Salin@net stations—records observations from GPS and GLONASS only. Consequently, in the main manuscript, we focus exclusively on the quality of the GPS constellation, while Supplementary File S1 reports the quality parameters for all additional constellations (GALILEO, GLONASS, and BEIDOU) acquired at each GNSS station.

The quality of GNSS data depends on several factors:

- Ratio of useful epochs;
- Multipath effect;
- Cycle slips;
- Signal-to-noise ratio;
- Skyplot observations density.

The first parameter we assessed was the station continuity. The continuity of the stations is described by the numbers of observations each day and the number of satellites and frequencies acquired during the operation time. Aspects such as electricity or the quality of the receiver may result in fewer than 2880 observations per day, which is the expected number of observations when the acquisition is performed with a sampling interval of about 30 s.

We evaluated the epochs counts on each day throughout the time period and calculated the ratio of useful epochs to total observations. We calculated this parameter as the ratio between the number of epochs with at least four satellites with dual frequency for an individual constellations over the total number of epochs. This metric is crucial, as an epoch is only valid if it includes data from at least two frequencies, which are essential for ionospheric delay correction.

A key parameter analyzed was the multipath effect. This effect results from GNSS signals reflecting off nearby surfaces or objects before reaching the receiver antenna. Both pseudorange and carrier-phase observations are affected by multipath, although for pseudorange the effect is higher [29]. To quantify this effect, we applied the ANUBIS algorithm, which uses a linear combination approach based on the methodology proposed by Estey & Meertens [27]. In this workflow, it is crucial to establish a minimum number of consecutive epochs to assess the multipath effect. We have determined a minimum requirement of 30 consecutive phase observations free of cycle slips. A cycle slip represents discontinuity in the phase measurement—specifically, an abrupt change in the number of phase cycles over time [30]. However, multiple values were tested, starting from 15 consecutive phase observations, which exhibited low variability in the expected multipath. It is essential to highlight that the multipath measurements also contain the signal noise effect, which cannot be further separated (see G-Nut/Anubis manual [31]).

As previously mentioned, multipath is influenced by cycle slips and signal noise. These two parameters can be estimated, enabling an assessment of their impact on signal quality. RINEX 2.xx format has less information about the signal respect to the RINEX 3.xx; hence, to obtain a more realistic comparison between all the GNSS stations, we compared cycle slips and signal-to-noise ratio only for the period in which also the ISAL station acquired RINEX 3.xx data (between 25 June 2024 and 22 November 2024).

A cycle slip can be caused by the receiver losing phase lock on the satellite signal, a power failure affecting the receiver software, or a malfunction of the satellite oscillator. Additionally, it can result from severe ionospheric conditions or, more commonly, obstructions that prevent the receiver from tracking the satellite signal. To handle such disruptions, various algorithms have been developed, and in this study, to analyze the amount of cycle slips, we employed the algorithm implemented in ANUBIS, as described by Zhao et al. [32].

Signal-to-noise ratio is a parameter that represents the strength of the signal with respect to the noise strength at a specific frequency, expressed in dB-Hz. The higher the signal-to-noise ratio, the higher the quality of the data obtained. This is an indicator of the antenna and receiver instrument performances [33], although it is also influenced by the vibration of the antenna [34]. The ratio is already in the RINEX data, as it is calculated by the receiver instantaneously. We show the signal-to-noise ratio for the L1, L2, and L5 GPS frequencies (and observations relative to the other constellations in Supplementary File S1), by assuming that all the receivers operate in the same way to calculate the parameter.

So far, we have listed all the parameters indirectly influenced by the geometry, the instrument, and the position of the GNSS station. To directly assess the quality of the positioning of the antenna, we used a combination of the navigation file, the satellite’s positions over time, and RINEX files. By processing these data, we calculated the elevation and azimuth of each observation relative to the GNSS station. Skyplots are a straightforward way to visualize the satellite visibility and identify potential obstructions that may affect signal reception during the entire acquiring period.

### 3.3. Data Processing

The raw observations were converted from the raw proprietary format to RINEX 3.02 format and processed by using the GAMIT/GLOBK 10.71 software suite [35], following the methodology described in Palano et al. [36], to estimate daily time series over the June 2023 to December 2024 time span. During the processing, only observations from the GPS constellation were considered. Although both GPS and GLONASS are available at all GNSS stations analyzed in this study, we restricted the analysis to GPS to avoid potential performance discrepancies arising from constellation-specific characteristics and to prevent introducing errors in the assessment of the GNSS stations quality (see Supplementary File S1 and Section 4.1). A cut-off angle of approximately 0° was adopted to include all available observations, and the Vienna Mapping Function (VMF1) [37] was applied to account for the tropospheric delay. Since the IS01, IS02, IS03, IS04, and IS05 stations have a limited temporal data span, the formal velocity uncertainties are likely underestimated. To account for temporally correlated noise not captured in the formal errors, we empirically scale the uncertainties by a factor of 5.

Residuals from the GNSS time series are evaluated using the Weighted Root Mean Square (WRMS), which provides a measure of the dispersion of the observations while accounting for their individual position uncertainties. Each residual is weighted by the inverse of its variance, so that more precise observations contribute more strongly to the final statistic. The WRMS is computed as follows:

(1) $$WRMS=\;\sqrt{\frac{\sum_{i}w_{i}r_{i}^{2}}{\sum_{i}w_{i}}}$$

(2) $$w_{i}=\frac{1}{\sigma_{i}^{2}}$$

where $r_{i}$ is the residual of observation i, $\sigma_{i}$ is its standard deviation, and $w_{i}$ is the corresponding weight. This metric provides a robust indicator of the overall quality of the GNSS residuals for each component.

We also downloaded meteorological data from Servizio Informativo Agrometeorologico Siciliano (SIAS, http://www.sias.regione.sicilia.it/ (accessed on 12 March 2025)), which took care of installing and collecting data from many meteorological stations. We chose the meteorological station “LENI” (see Figure 3 for location), within a distance of about ~300 m from IS01. We downloaded data during all the investigations time period in trying to understand the atmospheric daily mean temperature and daily temperature daily range trend with respect to the positioning errors.

To quantify the strength and direction of this potential relationship, we calculate the Pearson correlation coefficient (ρ) (Equation (3)) between the daily positioning measurements uncertainties on the north, east and up components and the mean daily temperature and Δ daily temperature:

(3) $$\rho =\;\frac{\sum_{i=1}^{n}\left(X_{i}-\bar{X})(Y_{i}-\bar{Y}\right)}{\sqrt{\sum_{i=1}^{n}{\left(X_{i}-\bar{X}\right)}^{2}}\sqrt{\sum_{i=1}^{n}{\left(Y_{i}-\bar{Y}\right)}^{2}}}$$

where $X_{i}$ and $Y_{i}$ are individual observations, $\bar{X}$ and $\bar{Y}$ are the mean values of X and Y, respectively, and n is the total number of observations.

### 3.4. Evaluating Network Geometry

A crucial part of this work involves evaluating the potential for identifying the behavior and associated risk of the Aeolian–Tindari–Letojanni and Sisifo–Alicudi fault systems in the near future. We applied the wavelet-based multiscale method outlined by Tape et al. [38] to derive a spatially continuous velocity field on the sphere from a set of unevenly distributed geodetic stations. This technique employs wavelets defined on increasingly finer grids, refining the spatial resolution only where the density of GNSS stations supports it. In practice, this means that high-resolution (short spatial distances) wavelets are used only in areas where the observation network is sufficiently dense. This adaptive approach ensures that the model captures the smallest geophysical signals that can be reliably resolved, based on the local distribution of observations [38,39].

The spherical wavelet analysis was performed using triangulated spherical grids, with a spatial extent between 14° E and 16° E in longitude and 37° N and 39° N in latitude, to obtain reliable results over the Aeolian Archipelago. The software used for the wavelet decomposition is provided by Tape et al. [38], available on https://github.com/carltape/surfacevel2strain.git (accessed on 12 March 2025).

Following the notation introduced by Tape et al. [38], the variable *q* denotes the wavelet order, which corresponds to a specific spatial resolution. For tectonic deformation studies, typical upper bounds for *q* fall between 8 and 10, representing spatial support of spherical wavelets of approximately 175 km to 44 km, respectively. We illustrate the highest wavelet order (*q*-scale) applied along the Sisifo–Alicudi and Aeolian–Tindari–Letojanni fault systems. In areas with a high density of GNSS stations, wavelets across the full range of scales (*q* = 1–12) can be used. Conversely, in regions with sparse station coverage, only longer wavelets associated (*q* = 1–7) are employed. The highest value reported by Tape et al. [38] is the order 12, representing a spatial wavelet of approximately 6 km.

We applied the algorithm using four different GNSS velocity field configurations

1. RING velocity fields;
2. RING + discrete GNSS [21] velocity fields;
3. RING + Salin@net velocity fields;
4. RING + discrete GNSS [21] + Salin@net velocity fields.

These four tests have been carried out to evaluate the potential of the Salin@net and the discrete measurements improvements.

## 4. Results

### 4.1. Quality Evaluation

The continuity of observations was assessed by examining the ratio of useful epochs to total observations to determine the quality of the data collection. No significant interruptions were observed for IS01, IS03, and IS05, even when compared to the observation counts from ISAL. However, IS02 experienced frequent interruptions between November 2023 and the end of March 2024 due to a faulty data cable, which was later replaced. As a result, the data remained consistent afterward. During the same period IS04 experienced a lack of L5 signal for wrong receiver settings, while during the period between the 4 and the 23 March 2024, there was no electricity, so the station was not operational. A significant reduction in the ratio of useful observations is observed between September 2023 and February 2024 for IS03. However, the values remain above 96%, ensuring a more than acceptable number of observations.

**Figure 4:**
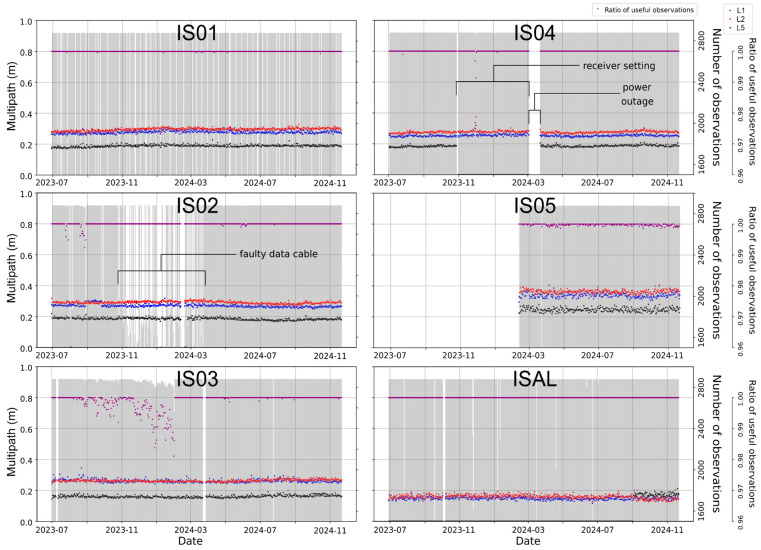
Analysis of the multipath effect, the number of epochs per day, and the ratio of useful epochs for each day for stations IS01, IS02, IS03, IS04, IS05, and ISAL.; multipath measurements are reported for L1, L2 and L5 frequencies of the GPS constellation. The time period depends on the station’s activity period.

The multipath effect, caused by signals reflecting off surrounding surfaces before reaching the receiver antenna, was analyzed to assess the reliability of GNSS measurements. The Guidelines for Continuous Reference Stations manual, published by the International GNSS Service (IGS) [14], states that an optimal multipath error value should consistently remain below or around 30 cm for all constellations (see Figure 4). The GPS L1 and L2 signals (mp1 and mp2 in Figure 4) consistently show values up to 0.28 cm for all the analyzed stations. In addition, the GPS L5 signal generally exhibits averaged values of 0.18 cm, with the exception of IS05, where the mean multipath value is approximately 0.25 cm, and IS04, which had an interruption on L5 frequency signal between November 2023 and March 2024. During the analyzed period, ISAL station recorded only L1 and L2 signals with mean multipath errors of ~0.18 cm, while L5 signal acquisition began in November 2024, and is therefore not available for the overall comparison.

The analysis of cycle slips revealed a similar pattern across all stations. Specifically, for the ISAL stations, the mean number of cycle slips during the considered period is ~1700. The best performance is observed at IS04, with consistently low values around 500 cycle slips. In contrast, IS01, IS02, and IS03 show similar behavior, with mean values of ~1500. The highest number of cycle slips is recorded at IS05, with values of ~2500 cycle slips.

**Figure 5:**
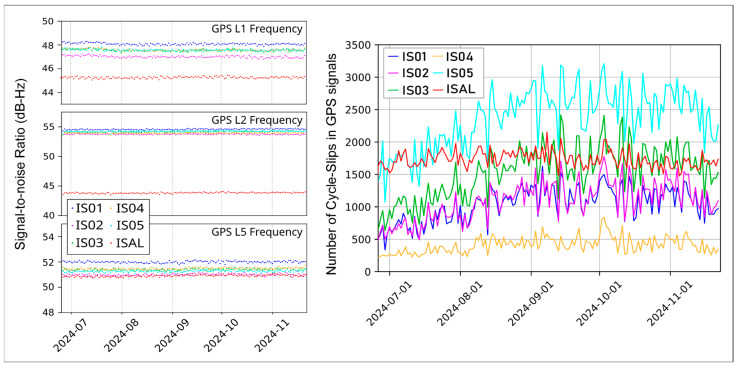
Signal-to-noise ratio and cycle slips on GNSS stations for the time period between the 25 June 2024 and the 22 November 2024. Period in which all the stations have RINEX 3.02 data.

All the GNSS stations across all considered frequencies exhibit stable signal-to-noise ratio values. Our reference station, ISAL, has a mean signal-to-noise ratio of approximately 45.3 dB in L1, 43.7 dB in L2, and 50.8 dB in L5. The newly installed GNSS stations demonstrate higher signal-to-noise ratio values compared to ISAL. Specifically, in the GPS L1 and L2 frequencies, the signal-to-noise ratio is higher by approximately 3 dB and 10 dB, respectively. In the L5 frequency, signal-to-noise ratio values range between 50.8 dB and 52 dB, with ISAL showing the lowest values in this frequency band as well (see Figure 5).

**Figure 6:**
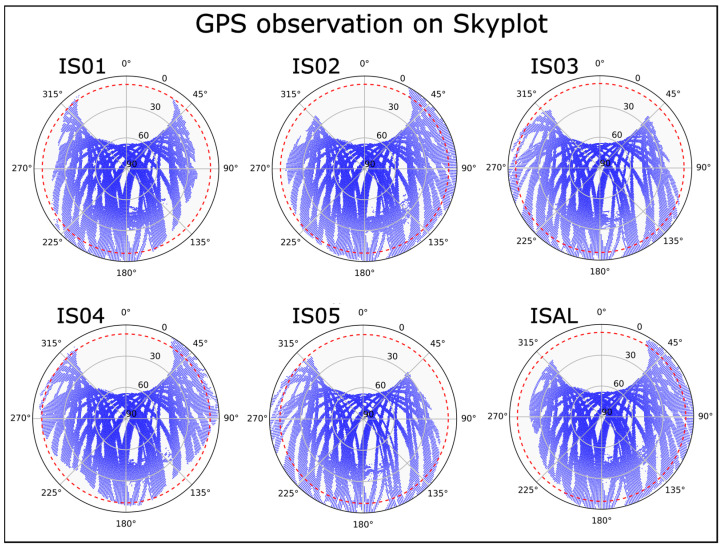
Skyplots for GPS observations during the period between 29 June 2023 and 22 November 2024. Red dashed lines represent the guideline-recommended threshold [14] of 10°.

Skyplots were utilized to identify potential obstructions affecting the GNSS stations. It is evident that topographic obstacles have slightly reduced the visibility of some of the new stations (Figure 6). However, visibility remains excellent when compared to ISAL station, as the shaded areas are consistently near the horizon, never exceeding 25°.

The quality parameters reported here have also been computed for all other constellations (GALILEO, GLONASS, and BEIDOU) and are presented in Supplementary File S1. The results for these additional constellations show a very similar behavior. The main differences observed are primarily attributable to the intrinsic characteristics of each constellation rather than to the performance of the individual GNSS stations.

### 4.2. Time Series

**Figure 7:**
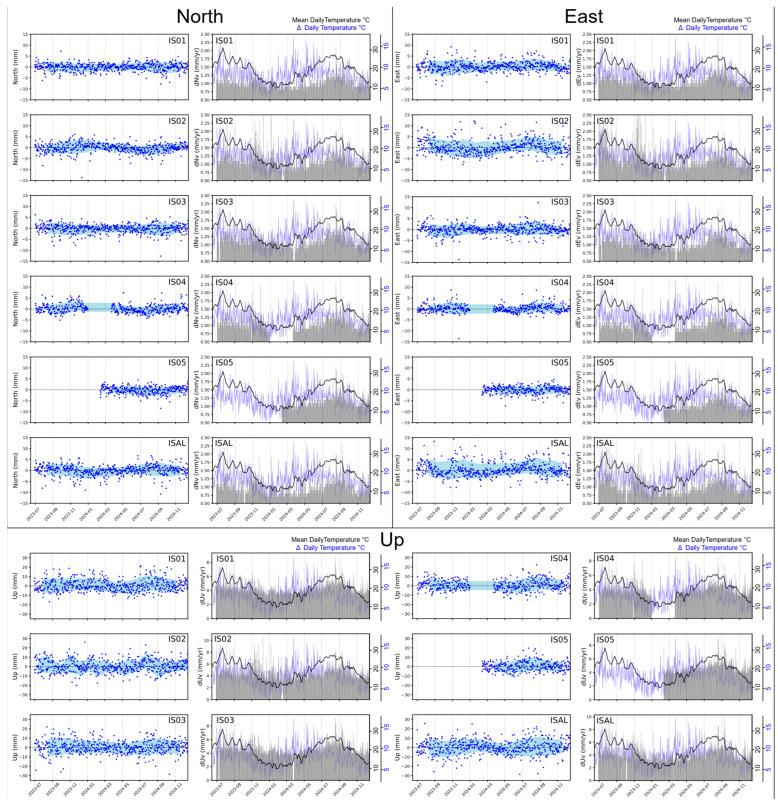
North–east–up positioning time series for Salin@net stations. In light blue is the area of standard deviation on a moving window of about 90 days. The histogram in gray represents the error associated with single positioning measurements. The black continuous line represents the mean daily temperature pattern of the LINA, while the blue continuous line (Δ daily temperature) represents the difference between the maximum and the minimum temperature in a day.

Estimated time series have been aligned to an ITRF2014 [40] reference frame during the computation with the GLOBK module of GAMIT. By using the GLOBK modules, all the velocity solutions have been aligned in the ETRF2014 [17] (Figure 2). In Figure 7, we reported the detrended time series in order to easily compare them. The detrended values, i.e., the linear velocities of the GNSS stations along with their estimated uncertainties, are reported in Table 2.

Along with the uncertainties in the linear velocity estimates, the WRMS (Equation (1)) was also calculated to quantify the noise in the time series. WRMS values range between 1.5 and 2.8 mm/yr for the horizontal components and between 5 and 6.7 mm/yr for the vertical component (Table 2). No significant patterns or anomalies were observed among the sites.

Daily positioning measurements uncertainties are on average ca. 1 mm for the horizontal components (north and east), while errors slightly increase by about 0.25–0.5 mm during the months of June, August, and September. This trend coincides with higher mean daily temperature values measured by LENI meteorological station (see Figure 3 for location). A similar pattern is observed in the vertical component (up), with mean uncertainties around 4 mm. During high temperatures (above 25 °C) periods, errors increase by at least 1 mm. Throughout the analyzed period, outliers are rare, and generally found in the east component. Given that most GNSS stations are installed on rooftops, we examine whether the observed trend may be attributed to the daily temperature range (Δ daily temperature in Figure 7), which can induce thermal effects on the buildings.

The results (Table 3) indicate a moderate correlation between the mean daily temperature and the daily positioning-measurement uncertainties (all components) for stations IS01, IS03, IS04, IS05, and ISAL, with values ranging from approximately 0.27 to 0.42. In contrast, station IS02 shows a weaker correlation, with values between ~0.1 and ~0.2. Conversely, values close to ~–0.2 to ~0, indicating a low or negligible correlation, are observed when considering the Δ daily temperature.

The velocity field, reported in Figure 3, has been rotated in ETRF2014 [17] to facilitate the interpretation of the deformation affecting the area. These velocities indicate a NNE-directed motion, with values ranging from approximately 3.5 to 4.7 mm/yr for the north component and from 0.79 up to 1.31 mm/yr for the east component.

### 4.3. Spherical Wavelet

**Figure 8:**
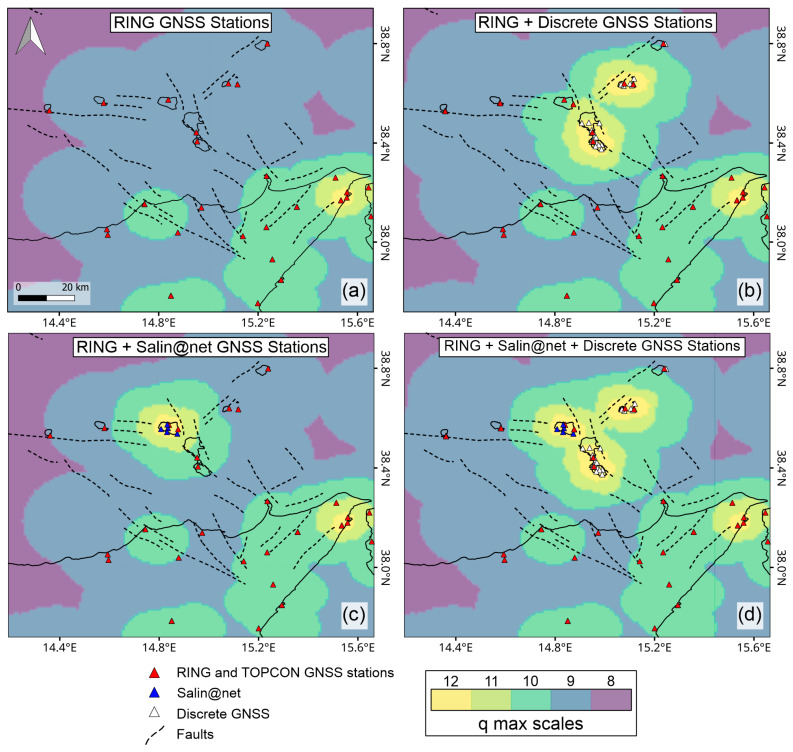
Wavelet spherical results by considering 4 velocity field configurations (see Section 4.3). The color map shows the maximum-q scale wavelet calculated by the [38] algorithm. Scale wavelets in this map are reported by orders from 12 up to 8, corresponding to spatial support from 6 km up to 44 km. Solid lines represent the coastline. In (**a**) the results only considering the RING stations [13], in (**b**) the RING stations [13] and the discrete GNSS measurements [21], in (**c**) both continuous RING [13] and Salin@net stations, in (**d**) the continuous RING [13] and Salin@net stations and discrete GNSS measurements [21].

In Figure 8, we can see all the interpolated wavelet spherical results on the Aeolian Archipelago area. Results are expressed relative to the maximum wavelet that covers each area.

In the first configuration (Figure 8a), considering only the RING continuous GNSS velocity field [13], the Aeolian–Tindari–Letojanni fault system mainly crosses areas where the maximum wavelet order (*q*) reaches approximately 9, corresponding to a spatial support of about 44 km. In this scenario the highest resolution (*q* ≈ 10) (spatial support ≈ 23 km) is achieved in the onshore segment of the Aeolian–Tindari–Letojanni fault system. By integrating the RING data with the discrete measurements (Figure 8c), the resolution increases significantly in localized clusters—particularly around Vulcano Island and the southern part of Lipari Island—where q values reach up to 12. In contrast, the Salina segment of the Aeolian–Tindari–Letojanni fault system, which represents a key area, attains a *q* value of approximately 10 (spatial support ≈ 23 km). A different pattern emerges in the third configuration (Figure 8b), where the combination of RING and Salin@net continuous GNSS stations leads to the highest q values (12) being concentrated around Salina Island. In this case, Lipari and Vulcano Islands exhibit lower maximum orders of around *q* = 10. The best spatial resolution along the Aeolian–Tindari–Letojanni fault system is achieved when all velocity fields are combined—RING [13], discrete GNSS measurements [21], and Salin@net. Under this configuration, very few areas fall below *q* = 9, and the fault is generally resolved at *q* ≈ 10 (spatial support ≈ 23 km) along most of its length.

Regarding the Sisifo–Alicudi fault system, the maximum order increases significantly, passing from 9 to 10, only in the cases in which we consider also the solution of Salin@net (Figure 8b,d) and specifically on the western side of Salina Island. These results indicate that the new GNSS network enhances the resolution along the Sisifo–Alicudi fault system as well, although additional monitoring stations are clearly required to better constrain the tectonic structures.

The results highlight the crucial role of the Salin@net network on Salina Island in achieving high-resolution strain evaluations onshore, as well as improving the resolution offshore, thereby enhancing the capability to detect the presence of the Aeolian–Tindari–Letojanni fault system. Moreover, the inclusion of high-density measurements—such as the discrete GNSS measurements—proves essential for characterizing the behavior of the fault along its entire extent.

## 5. Discussion and Conclusions

The findings of this work indicate a very promising overall data quality. The multipath effect remains within optimal limits at all Salin@net stations, with values below 30 cm across all frequencies. This threshold corresponds to the optimal value recommended by the guidelines of Bradke et al. [14]. Regarding signal continuity, we infer that the quality is acceptable as the minimum required parameter for the useful epoch ratio is 0.95 (95%) by the previously mentioned manual. The visibility of the GNSS stations was evaluated through skyplots, which show that topographic obstacles locally reduce satellite visibility. Although some stations exhibit small, shaded sectors with elevation angles exceeding 10°, these areas are limited in extent, particularly given the complex topography of Salina Island. The number of cycle slips was necessarily compared with the ISAL reference station. Observing the average value of ~1700 for total cycle slips, we can infer that these are from the stations IS01, IS02, and IS04, while IS03 exceeds this value only in 5% of the cases. IS05 records the highest number of cycle slips during the operational period. Despite their occurrence, these interruptions can be efficiently corrected during post-processing. The analysis of the signal-to-noise ratio indicates that the RING reference station, ISAL, exhibits higher noise levels compared to the newly installed Salin@net GNSS stations.

The positioning time series analysis allows for a more detailed evaluation of whether issues such as cycle slips or partial obstructions of the instrument significantly impact the data or can be reliably corrected during processing. The positioning time series graphs (Figure 7) of the processed stations confirm that the signal has been effectively corrected, with velocity error values remaining comparable across all stations. The IS05 station shows slightly higher error values in velocity measurements, but this issue is due to its still limited acquisition period, with an operational time of less than one year. We suggest that cycle slips have been appropriately corrected by the processing, as long as their number remains within a range that does not pose a significant issue. The Pearson correlation coefficients (see Equation (3) and Table 3) between daily positioning errors and the mean daily temperature near the GNSS stations highlights an imperfect tropospheric correction during the processing workflow rather than an issue with the quality of the stations installed. To support this analysis, we also investigated whether the buildings might be affected by a non-negligible daily thermal effect by comparing the daily positioning uncertainties in the north, east, and up components with the daily Δ temperature (see Figure 7). In this case, only a low correlation was observed, clearly indicating that the GNSS stations can be considered stable with respect to daily thermal variations. In general, one would expect differences in the correlation coefficients between GNSS stations installed on rooftops and those on bedrock if such thermal effects were significant; however, no such differences are observed. This is further confirmed by the comparable Weighted Root Mean Squares (WRMS) values (Equation (1) and Table 2) observed for all the GNSS stations.

By considering both reference frames (ITRF2014 and ETRF2014), the difference in the velocity among the Salin@net and ISAL stations positioned on Salina Island is only about 1 mm/yr. While no significant velocity differences are currently apparent among the stations, it is crucial to note that the available data remains very limited. It is well known that the length of the time series is crucial for the accuracy of the estimation of the linear velocities [41]. Different authors have attempted to determine the minimum number of years required for a reliable estimation [39]. In the best-case scenario, we need to wait at least 2.5 years [41] before considering the linear velocity measurements reliable. Results of the spherical wavelet analysis show how the inclusion of the Salin@net GNSS network significantly enhances the resolution at which the Aeolian–Tindari–Letojanni fault system can be identified and characterized in the near future. Furthermore, the inclusion of the high-density GNSS measurements, whether continuous or discrete, on Lipari and Vulcano islands clearly highlights their critical role in the geodetic signal detection. Indeed, these data are essential to ensure full coverage along the entire length of the Aeolian–Tindari–Letojanni fault system and to accurately describe the deformation processes currently acting on the area. Significant improvements were also observed for the eastern portion of the Sisifo–Alicudi fault system, although additional GNSS stations on Alicudi and Filicudi Islands would be needed to improve detection along the entire fault length.

We are confident that the installation quality of the Salin@Net GNSS stations—evaluated against international guidelines [14] and the standards of continuous RING stations—is optimal. The network is thus well-suited to deliver high-quality geodetic data essential for investigating crustal deformation processes in the Aeolian Archipelago. In the near future, the continuous data stream from Salin@Net is expected to provide valuable insights to better quantify and constrain the activity of the Aeolian–Tindari–Letojanni and Sisifo–Alicudi fault systems, thereby improving our understanding of their role in the regional geodynamics and contributing to seismic hazard mitigation efforts.

Although preliminary, the horizontal GPS velocity field at Salina Island (Figure 3) shows negligible lithospheric shortening along the NNE–SSW direction. Moreover, the velocity gradient between eastern and western sectors does not decrease northward, arguing against Salina acting as a transitional zone between the western compressional and central–eastern transtensional–extensional regimes. These observations suggest that the both surface expressions of the Sisifo–Alicudi and Aeolian–Tindari–Letojanni fault systems are likely offshore, highlighting the importance of integrating onshore GNSS with marine geophysical data to fully capture the regional deformation pattern.

## Figures and Tables

**Table 1:** Geographic coordinates and instrumental information about GNSS stations from Salin@net and ISAL from the RING network [13].

Station Name	Longitude	Latitude	Receiver	Antenna	Sampling Interval	Constellations	Data Format	Acquisition Start Date
IS01	14.83062	38.56369	STONEX SC600+	STONEX STXSA1100	30 s	GPS, GALILEO, GLONASS, BEIDOU	RINEX 3.02	29 June 2023
IS02	14.86844	38.54165	STONEX SC600+	STONEX STXSA1100	30 s	GPS, GALILEO, GLONASS, BEIDOU	RINEX 3.02	30 June 2023
IS03	14.82723	38.54822	STONEX SC600+	STONEX STXSA1100	30 s	GPS, GALILEO, GLONASS, BEIDOU	RINEX 3.02	1 July 2023
IS04	14.82806	38.57836	STONEX SC600+	STONEX STXSA1100	30 s	GPS, GALILEO, GLONASS, BEIDOU	RINEX 3.02	2 July 2023
IS05	14.80305	38.56038	STONEX SC600+	STONEX STXSA1100	30 s	GPS, GALILEO, GLONASS, BEIDOU	RINEX 3.02	12 February 2024
ISAL	14.87171	38.55959	LEICA (Switzerland) GR25	LEICA (Switzerland) LEIAR20	30 s	GPS, GLONASS	RINEX 2.11-3.02	30 October 2018

**Table 2:** GNSS velocities in ITRF2014 reference frame [17] and in ETRF2014 [17] for the north, east, and up components, together with the Weighted Root Mean Square (WRMS) (Equation (1)) of the time series in Figure 7.

Station Name	North Velocity ITRF2014 (mm/yr)	North Velocity ETRF2014 (mm/yr)	North WRMS	East Velocity ITRF2014 (mm/yr)	East Velocity ETRF2014 (mm/yr)	East WRMS	Up Velocity (mm/yr)	Up WRMS
IS01	18.84 ± 0.8	3.65 ± 0.8	1.6	23.8 ± 1.05	0.97 ± 1.05	2.1	−1.43 ± 2.45	5.9
IS02	18.7 ± 0.9	3.51 ± 0.9	1.8	24.15 ± 1.4	1.31 ± 1.4	2.8	−0.79 ± 2.45	6
IS03	18.79 ± 0.9	3.6 ± 0.9	1.8	23.73 ± 1.0	0.9 ± 1.0	1.9	−0.14 ± 3.2	6.3
IS04	19.87 ± 0.85	4.68 ± 0.85	1.7	23.61 ± 0.75	0.79 ± 0.75	1.5	0.04 ± 2.5	5
IS05	18.98 ± 1.65	3.78 ± 1.65	1.6	24.13 ± 1.6	1.31 ± 1.6	1.6	0.04 ± 5.2	5
ISAL	18.53 ± 0.20	3.34 ± 0.20	2	23.86 ± 0.29	1.03 ± 0.29	2.8	−1.67 ± 0.69	6.7

**Table 3:** Pearson correlation coefficient (ρ) for Salin@net and ISAL GNSS stations.

Station Name	ρ Mean Daily Temperature/dN	ρ Mean Daily Temperature/dE	ρ Mean Daily Temperature/dU	ρ Δ Temperature/dN	ρ Δ Temperature/dE	ρ Δ Temperature/dE
IS01	0.395	0.439	0.416	−0.054	−0.026	−0.041
IS02	0.098	0.208	0.113	−0.23	−0.128	−0.221
IS03	0.3568	0.4046	0.37	−0.114	−0.088	−0.102
IS04	0.272	0.284	0.289	−0.06	−0.053	−0.063
IS05	0.3447	0.421	0.361	−0.195	−0.142	−0.196
ISAL	0.317	0.334	0.335	−0.078	−0.077	−0.076

## Data Availability

The original contributions presented in this study are included in the article/Supplementary Material. Further inquiries can be directed to the corresponding author(s).

## Supplementary Materials

The following supporting information can be downloaded at https://www.mdpi.com/article/10.3390/s25237362/s1. File S1: quality parameters for the Salin@net and ISAL stations for the constellation GALILEO, GLONASS and BEIDOU.

## Abbreviations

The following abbreviations are used in this manuscript:

ATLFS	Aeolian-Tindari-Letojamni Fault System
SASF	Sisifo–Alicudi Fault System
mp	Multipath values
