# Supplementary material for: Improving Geodetic Monitoring in the Aeolian Archipelago: Performance Assessment of the Salin@net GNSS Network
Source: Sensors (Basel). 2025 Dec 3;25(23):7362. doi: 10.3390/s25237362 (PMC12694197; doi:10.3390/s25237362)
Supplement: Supplementary file 1 [file sensors-25-07362-s001.zip › sensors-3982651-supplementary.pdf]

Supplementary file S1

This supplementary file contain the analysis on multipath, number of observations, signal-to-noise ratio and cycle-slips for GNSS sites of Salin@net (IS01, IS02, IS03, IS04, IS05) for the constellations GALILEO, GLONASS and BEIDOU, while the analysis of ISAL station only for the GLONASS constellation.

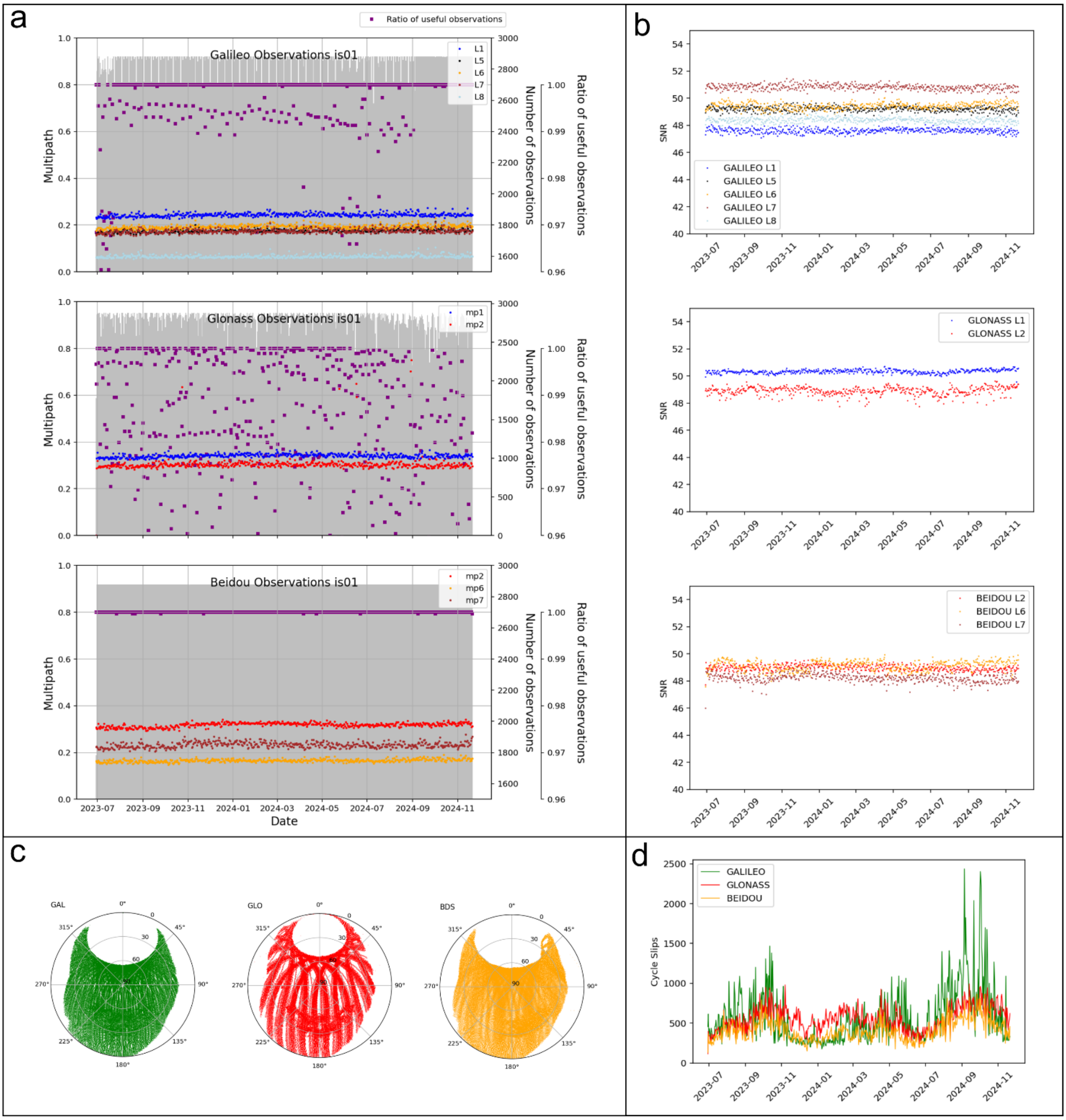

Figure 1: Quality estimation for IS01 GNSS station. a) Multipath analysis for the Galileo, Glonass and Beidou constellations. Multipath values, expressed in meters, are shown as points coloured according to the observation frequency: L1 (mp1, blue), L2 (mp2, red), L5 (mp5, black), L6 (mp6, orange), L7 (mp6, brown), L8 (mp6, azure). With a grey area is reported the number of observations. The grey-shaded area indicates the daily number of observations, with a maximum of 2880 observations per day at a 30-second sampling rate. Purple points indicate the ratio of useful observations, defined as the ratio between the number of epochs with at least

4 dual-frequency satellites and the total number of epochs. b) Signal-to-noise ratio (SNR) for the Galileo, Glonass and Beidou constellations, measured in dbHz. Points are coloured by observation frequency, consistency with panel a). c) Skyplot density of observations for the entire period analyzed in the main manuscript. Observations are coloured based on constellations: Galileo (green), GLONASS (red) and Beidou (orange) d) Total number of daily Cycle-slips. Coloured coloured by constellations, consistency with panel c).

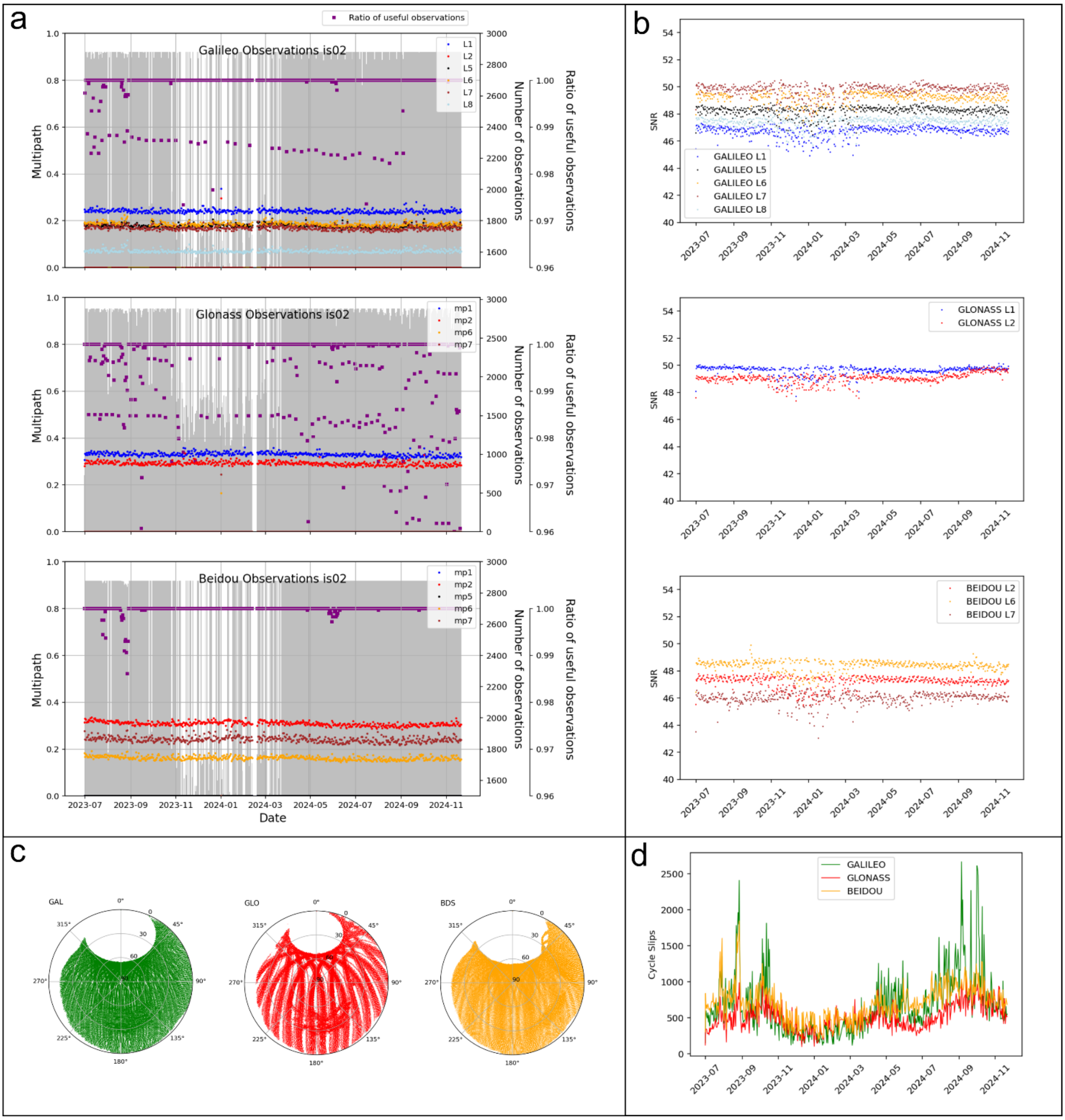

Figure 2: Quality estimation for IS02 GNSS station. Panels a), b), c) and d) description same as Figure 1.

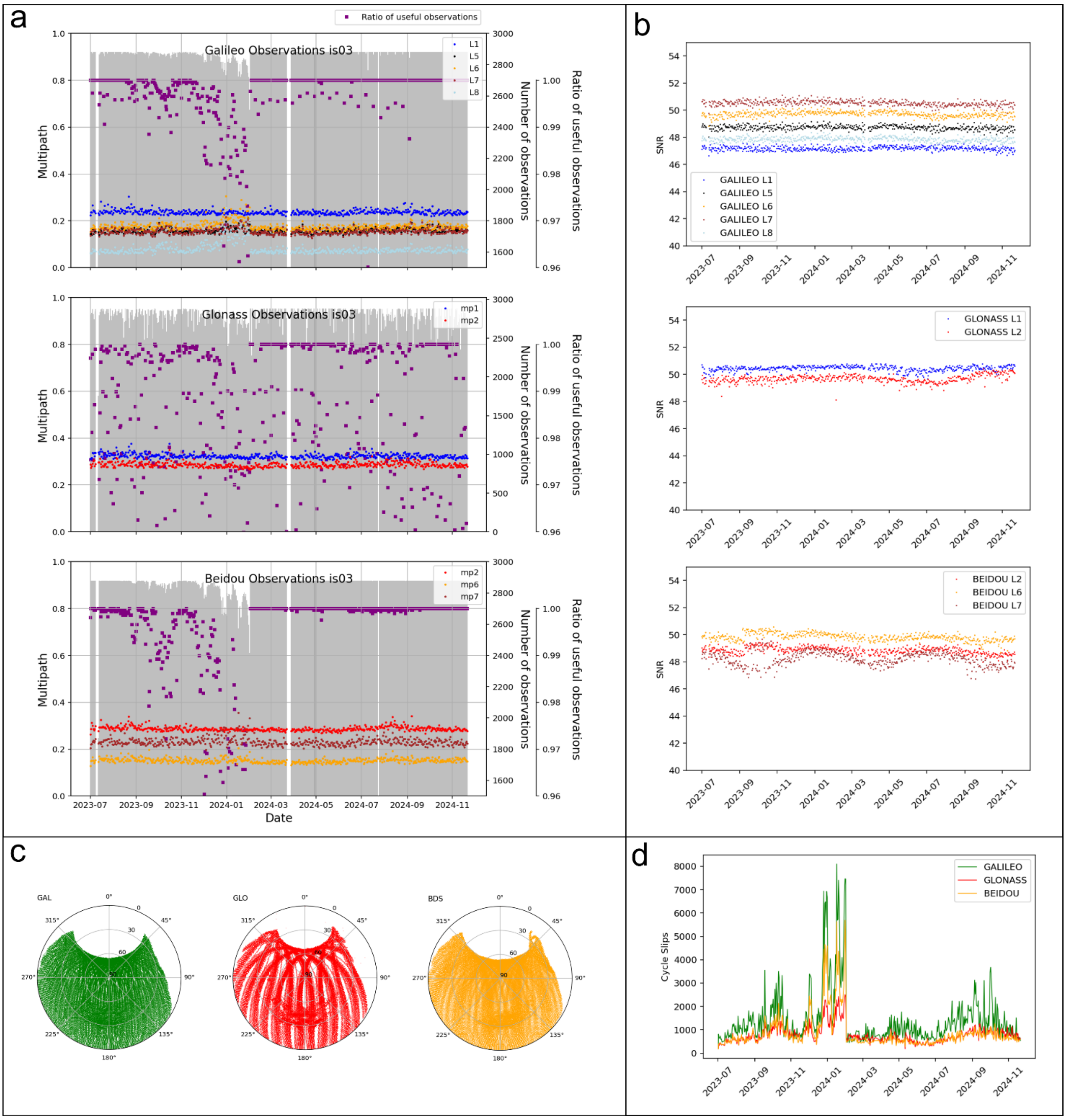

Figure 3: Quality estimation for IS03 GNSS station. Panels a), b), c) and d) description same as Figure 1.

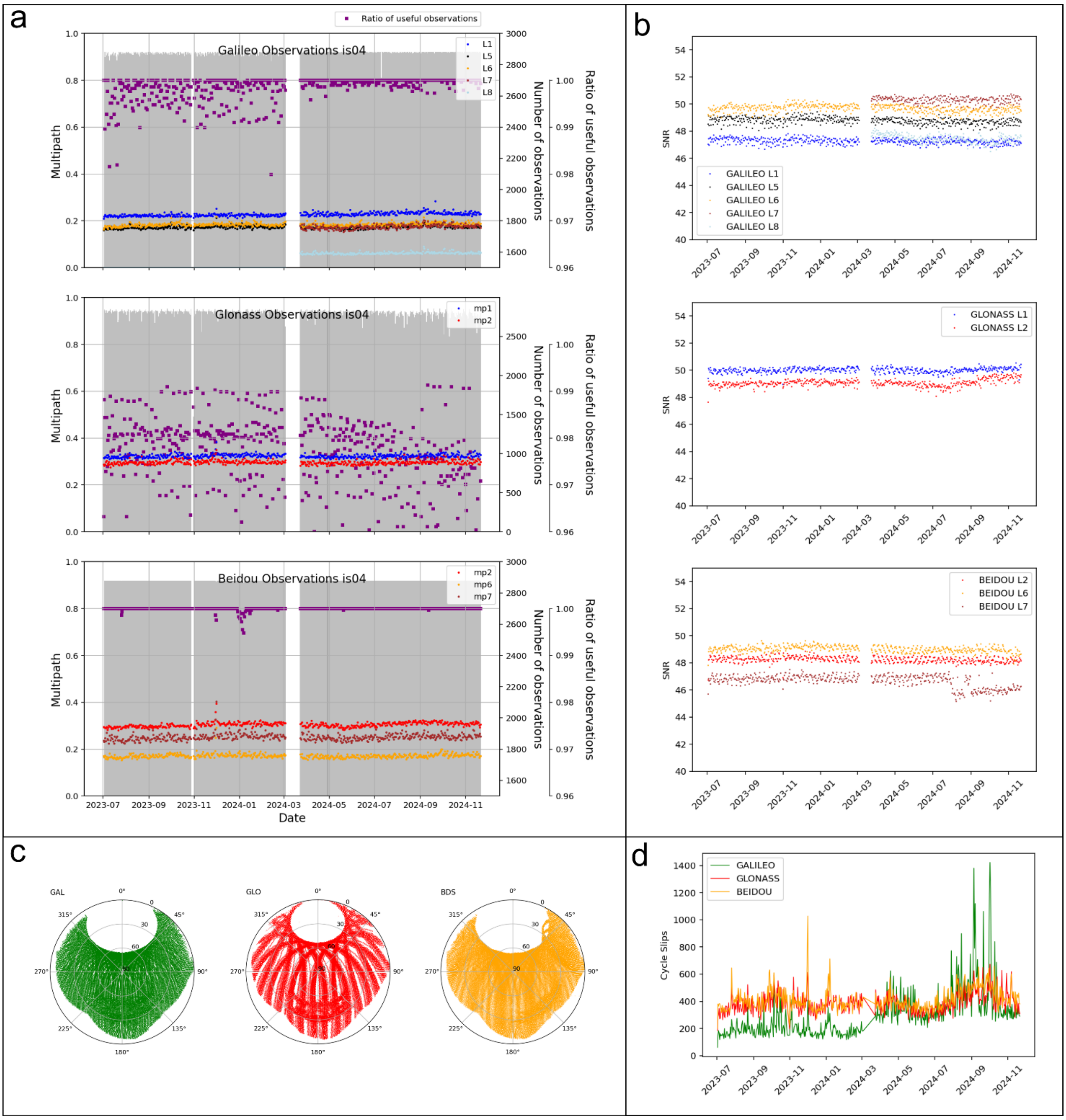

Figure 4: Quality estimation for IS04 GNSS station. Panels a), b), c) and d) description same as Figure 1.

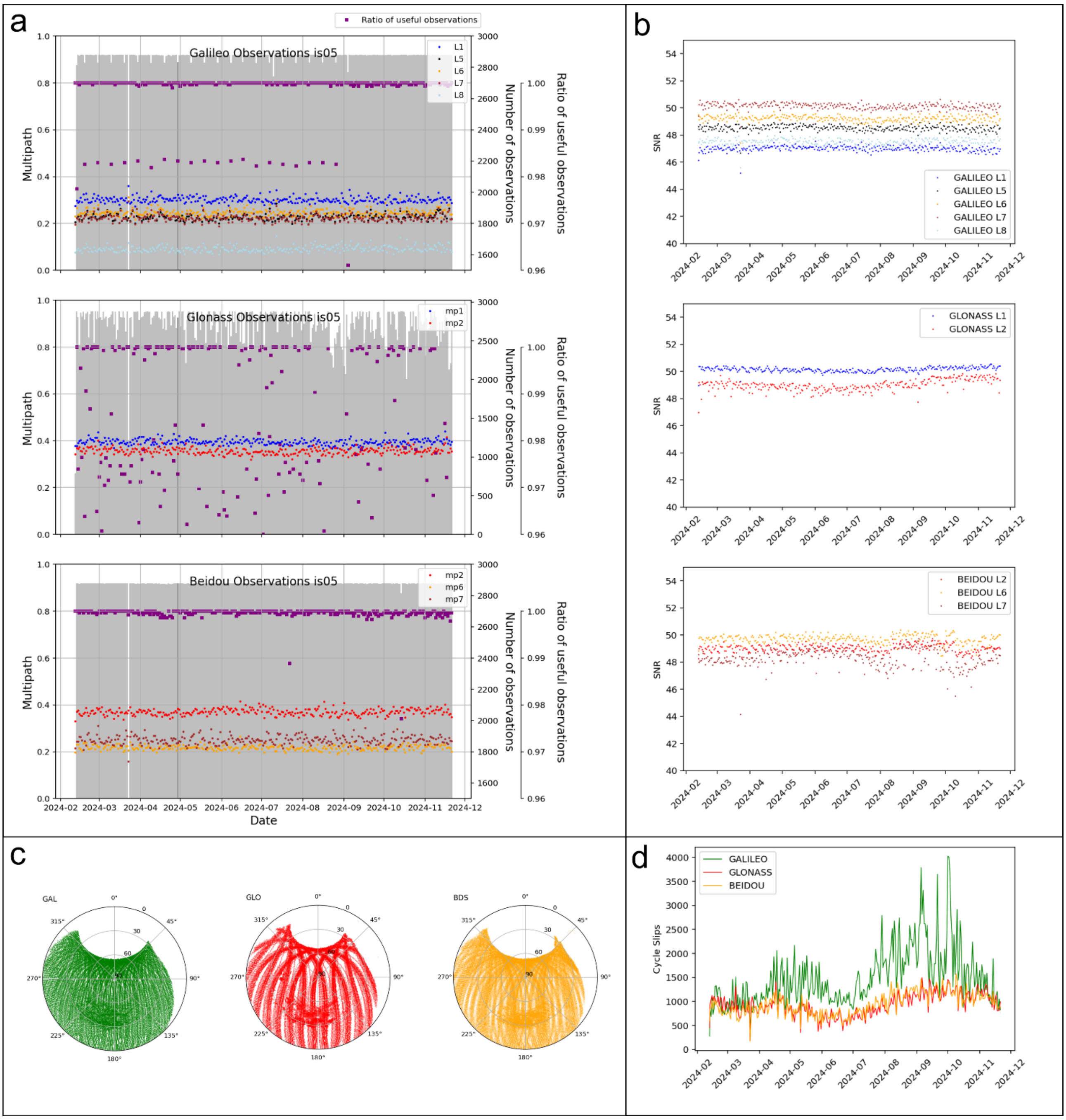

Figure 5: Quality estimation for IS05 GNSS station. Panels a), b), c) and d) description same as Figure 1.

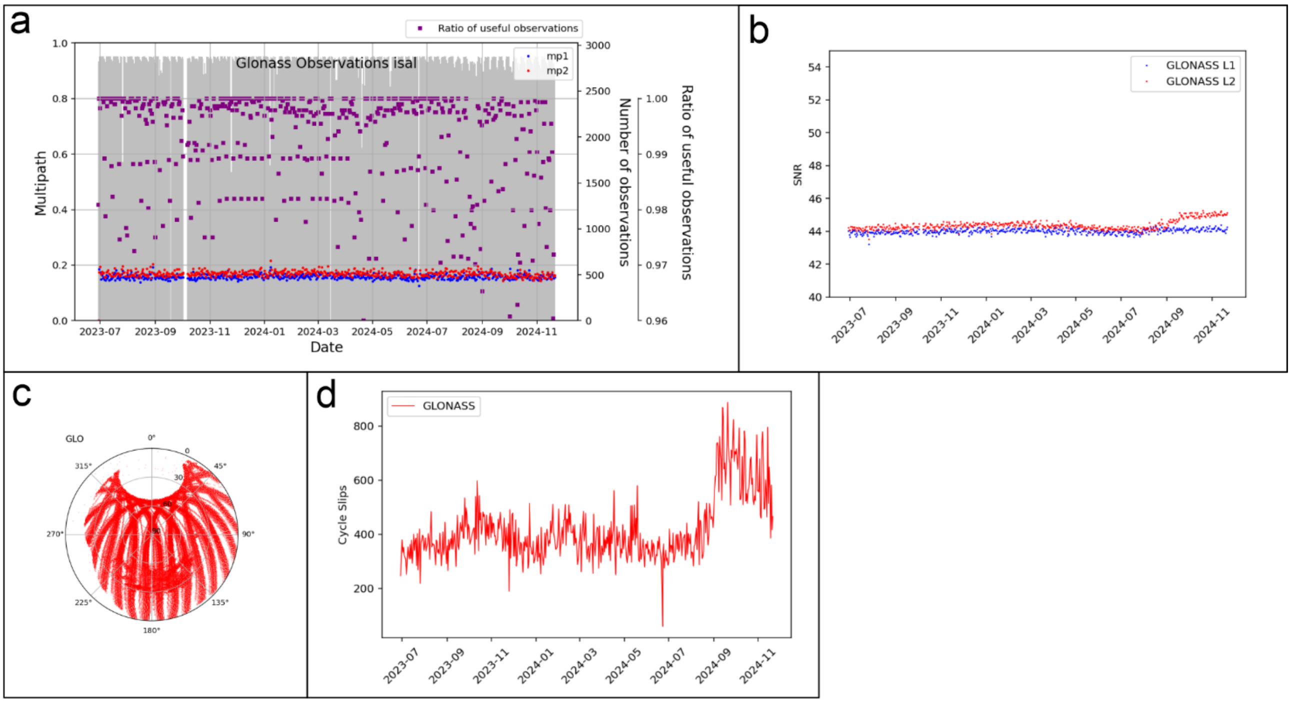

Figure 6: Quality estimation for ISAL GNSS station. a) Multipath analysis for the Glonass constellation. Multipath values, expressed in meters, are shown as points coloured according to the observation frequency: L1 (mp1, blue), L2 (mp2, red). With a grey area is reported the number of observations. The grey-shaded area indicates the daily number of observations, with a maximum of 2880 observations per day at a 30-second sampling rate. Purple points indicate the ratio of useful observations, defined as the ratio between the number of epochs with at least 4 dual-frequency Glonass satellites and the total number of epochs. b) Signal-to-noise ratio (SNR) for the Glonass constellation, measured in dbHz. Points are coloured by observation frequency, consistency with panel a). c) Skyplot density of observations from Glonass constellation for the entire period analyzed in the main manuscript. Observations are coloured in red. d) Total number of daily Cycle-slips.
